# Almajiri health; a scoping review on disease, health literacy and space for participatory research

**DOI:** 10.1371/journal.pgph.0001641

**Published:** 2023-10-11

**Authors:** Muzzammil Imran Muhammad, Amna Hassana Abdulsalam, Fadhina Petit-Clair, Amber Acquaye, Autumn Nobles, Brian Fleischer, Chigoziri Konkwo, Yusuf Ransome, Elijah Paintsil

**Affiliations:** 1 Yale School of Medicine, New Haven, Connecticut, United States of America; 2 National Agency for the Control of AIDS, Abuja, Federal Capital Territory, Nigeria; 3 Yale University, New Haven, Connecticut, United States of America; 4 Department of Social and Behavioral Sciences, Yale School of Public Health, New Haven, Connecticut, United States of America; 5 Department of Pediatrics, Section of Infectious Diseases and Global Health, Yale University School of Medicine, New Haven, Connecticut, United States of America; VART Consulting PVT LTD, INDIA

## Abstract

**Introduction:**

*Almajirai* are male children in Northern Nigeria and Southern Niger who study Islam in the *almajiranci* system. Almajiranci has been associated with non-participation in formal education, abuse, poverty, and underdevelopment. However, the peer-reviewed literature around health among almajirai remains limited. We conduct a scoping review around almajiri health to synthesize evidence for health problems, draw links between findings, identify research gaps, indicate areas for intervention, and assess participatory approaches in this literature.

**Methods:**

We searched the academic literature for articles concerning almajiri heath using a framework integrating the biopsychosocial and socio-ecological models of health. We included articles in English and French published between 2000 and 2022. For each study we collected information regarding authorship, study year and location(s), study design and aims, sample characteristics, findings, and almajiri participation in research design, execution, interpretation and dissemination.

**Results:**

Of 1,944 studies, 17 were found relevant for data extraction. These included 14 cross-sectional studies, 2 descriptive articles, and one case-control study. All were conducted in Nigeria, though one included Nigerien almajirai. No study engaged almajirai in participatory roles. Domains evaluated included infectious disease (10 studies), oral health (2 studies), workplace injury, nutrition, health status, health determinants, and mental health (1 study each). Almajirai included ranged from 3 to 28 years old. Included studies found high rates of malaria, intestinal parasitosis, urinary tract infection, N. meningitidis, and occupational injury among almajirai. Studies comparing almajirai to controls found significantly higher rates of cholera, urinary schistosomiasis, and psychiatric disorders, lower levels of rabies awareness and poorer oral hygiene among almajirai (p<0.05). One study, concerning nutrition, described an intervention to improve almajiri health, though did not provide health outcomes for that intervention.

**Conclusion:**

We find that the literature around almajiri health has concerned a broad range of domains, though the number of studies within each domain remains limited. We further note limitations in the geographic scope of this literature, interventions to improve almajiri health, and the consideration of demographic features, like age, that may influence almajiri health. We stress the need for further study in these areas, and for participatory approaches, which may be more likely to effectively improve almajiri health.

## Introduction

*Almajirai* (singular: Almajiri) are male children who leave the care of their parents to learn the Qur’an and study Islam under the tutelage of a *Mallam* at a *Tsangaya*. *Almajiranci* refers to the system of education based on this relationship between almajiri and mallam, and which was the dominant mode of education in precolonial Hausaland–a region encompassed in Northern Nigeria and Southern Niger, and with some of the world’s worst measures of human development and population health [1–4]. The underperformance of public education and limited access to the means to afford private secular education in this region has meant that this system remains widespread and popular in both countries [5].

The word almajiri is a Hausa term that derives from the Arabic *al-muhājirūn (*المهاجرون), meaning migrants, and alludes to the migration of Muhammad (ﷺ) and his followers from Mecca to Medina in the early history of Islam [6]. This reflects the experience of migration from parental home to Tsangaya that is a core feature of almajiranci [5]. At their Tsangayu, almajirai live under and receive Islamic education from their Mallamai, and may also take on employment at local households and businesses. Almajirai number in the millions in Nigeria alone, and are ubiquitous throughout urban Hausaland, often seen living, sleeping and working on the street [5]. The majority of almajirai are children, many of whom enter the system at ages as young as three years old, though some almajirai are considerably older, and may continue education at a Tsangaya into late adolescence and even early adulthood [5, 7].

Among children, street life has been associated with a variety of poor outcomes, including greater exposure to abuse and violence, poorer mental health, malnutrition and greater risk of communicable disease, though many of these findings are specific to certain populations of street children [8, 9]. A substantial body of literature has considered these dynamics among almajirai, demonstrating their exposure to considerable harm, including exposure to the elements and vectors of communicable disease, occupational hazard in the odd jobs they may take on in urban communities, limited access to complete nutrition, and higher risk of involvement in motor vehicle accidents [5, 10]. In the context of increasing militancy and violence in much of the Sahel, including Hausaland, some have argued that the social marginalization almajirai experience may render them vulnerable to recruitment on the part of criminal organizations [11, 12]. Despite a lack of evidence demonstrating a higher likelihood of such recruitment among almajirai, these concerns still persist, contributing to some degree of moral panic around almajiranci [13].

An increasingly prominent research concern has been the health of almajirai, with findings that suggest increased risk of cholera, urinary schistosomiasis, and identifiable psychiatric diagnoses among almajirai than in control populations [14–16]. The COVID-19 pandemic has also contributed to some distress around almajiri health, as their perceived susceptibility to infectious diseases and high geographic mobility have caused them to be seen as a population at heightened risk of viral infection and transmission [17]. Despite this, the peer-reviewed literature around health among almajirai remains limited. Stakeholders involved in research and policymaking around almajiranci have noted a lack of literature around the health of almajirai, as well the challenges that this lack of literature create for intervention and partnership towards supporting these communities [16].

Lastly, almajirai, street children and children more broadly have often been portrayed in the literature as “vulnerable, incompetent and … powerless in society” [18]. These portrayals strip these children of their agency and lead to the exclusion of their voices from work intended to provide them support. Attention has been increasingly paid to these children’s agency and their roles as “social actors” capable of effective engagement with their surroundings to protect their own interests and wellbeing. That many almajirai view their involvement in almajiranci as a temporary hardship endured for the sake of gaining knowledge, developing social resilience, and establishing strong spiritual and moral foundations, reflects this type of engagement [5]. Some work has engaged almajirai in participatory roles, and has found that, given this type of opportunity, almajirai effectively and enthusiastically identify and advocate for their concerns and interests. These opportunities, however, are not always without complication, with collaboration impacted by concerns around material compensation and exploitation [19]. This demonstrates that participatory approaches hold great promise for engagement with almajirai, as has been shown for other marginalized groups, where participatory approaches “promote equitable engagement of residents, community-based organizations, governmental and service-providing agencies, and academic institutions in the process of designing and implementing efforts” to improve these groups’ health, but also require care to ensure that all parties’ interests are protected in participatory work and its outcomes [20]. This type of approach may be of particular importance in contexts like Hausaland, where feelings of distrust for modern medical institutions are widespread and have had fatal consequences [21, 22].

Here, we present a scoping review of the academic literature regarding the health of almajiri communities.

**Glossary**

Selected terms used with high frequency in this manuscript, all of which derive from the Hausa language

**Almajiranci**: A system of education prevalent in *Hausaland* whereby male children leave their familial homes to study Islam and the Qur’an under the tutelage and guardianship of a *Mallam* in a *Tsangaya*.

**Almajiri** (plur. **Almajirai**): A male child engaged in *Almajiranci*. Derives from the Arabic *al-muhājirūn* (المهاجرون), meaning migrants, and alludes to the migration of Muhammad (ﷺ) and his followers from Mecca to Medina in the early history of Islam.

**Hausa**: A West African ethnic group native to *Hausaland* and speaking Hausa, an Afroasiatic language. The largest ethnic group in both Nigeria and Niger by population.

**Hausaland**: The region of West Africa to which the *Hausa* people are indigenous and/or where Hausa is a predominant language, largely located in Northern Nigeria and Southern Niger.

**Mallam** (plur. **Mallamai**): A male adult responsible for the care and education of *almajirai* in a *Tsangaya*. Also used as a generic honorific in the *Hausa* language, akin to ‘Mister’ in English. Derives from the Arabic *Mu’allim* (معلم).

**Tsangaya** (plur. **Tsangayu**): The schools wherein *Almajirai* learn and often also live.

## Methods

### Study design, rationale and aims

Scoping reviews are "preliminary assessments of the potential size and scope of available research literature” which help “identify the nature and extent of research evidence” [23]. Scoping reviews are particularly appropriate "when a body of literature has not yet been comprehensively reviewed, or exhibits a large, complex, or heterogeneous nature not amenable to a more precise systematic review” [23]. Scoping review designs have been of value in health research at the population and community levels, and in a variety of contexts [24–26]. A strength of this research design has been its ability to use evidence of various types to provide detailed reflections on current knowledge and help map areas for research, partnership and intervention.

We use a scoping review on the health of almajiri populations to leverage this strength, and help better define this research space, following guidelines around the conduct of scoping reviews outlined in literature [27–29]. The question that motivates this review is the following: “What is known about the health of almajirai, what interventions have been considered or implemented to help improve health in almajiri communities, and to what extent has the literature around almajiri health engaged almajirai and their communities as participants?”. Our specific aims are to

- Synthesize evidence for specific health findings among almajirai
- Draw links between related health findings for almajirai
- Identify specific gaps in the literature around health in almajiri populations
- Indicate areas for potential intervention in improving health in almajiri populations
- Assess if and how almajirai and their communities have been engaged as participants in research and interventions around almajiri health

### Geographic scope

We consider almajiri communities throughout historic Hausaland, in Northern Nigeria and Southern Niger. This contrasts with other approaches to almajiranci seen in literature, which have mostly concerned communities and populations in Northern Nigeria, the more populous of Hausaland’s two regions. We take our approach to represent the fullest possible breadth of almajirai and their experiences, and in recognition of generally porous borders of the West African Sahel, which have fostered a myriad many transnational communities throughout Hausaland and the Sahel more broadly [30, 31].

Despite the presence of Quranic schools and other institutions that may resemble almajiranci throughout many parts of Muslim West Africa, we do not extend our search to settings beyond Hausaland. This reflects differences in history, culture, religious perspective, political dynamics and economic circumstance that may have profound impacts on the shapes of these institutions and the experiences of their students [32, 33]. While these same differences also exist within Hausaland, reflecting distinct experiences of colonization and independence, the profound linguistic, cultural and personal connections within this region, as well as evidence demonstrating the movement of almajirai between Nigeria and Niger, support an approach that considers almajiri communities in both countries [34].

### Operationalizing ‘almajiri health’

We organize our conceptualization of health using Engel’s biopsychosocial formulation, which posits that health exists under the influence of biological factors, such as age, gender and physiology; psychological factors, including mental health and individual understandings and experiences around health; and social factors, such as access to care and social supports, as well as systems-level social dynamics [35].

We supplement this model with the social ecological model, which uses systems theory to argue that communities exist in interdependent and mutually-shaping relationships with their environments that resemble ecological relationships observed in nature. These social-ecological relationships may be reciprocally reinforcing, but also considerably multifaceted, creating space for agency through individual differences in these relationships [36]. This framework has been used to enrich use of the biopsychosocial model elsewhere in the literature around the health of marginalized populations [25].

We integrate these frameworks into a model that places health outcomes among almajirai under the influence of multidirectional relationships between biologic factors, including medical history and specific disease risks, social factors, such as shelter, employment, education and income, and psychological factors, such as almajirai’s own understanding of their role and the ways they engage society, effectively and not, to protect and advance their own interests, aspirations and wellbeing, enabling a thorough approach to the question of almajiri health.

### Inclusion criteria, search strategy & data extraction

We obtained English- and French- language studies in the academic literature regarding almajiri health, as previously conceptualized, in Nigeria and Niger, and published between January 1, 2000, and September 16, 2022. Our search strategy was developed through consultation with an academic librarian. Databases used to perform our search were MEDLINE, Embase, OVID Global Health, Scopus, Web of Science and EBSCO’s Africa-Wide Information Database. Though we considered performing searches on Google Scholar to capture studies in the grey literature, this database was ultimately excluded for concerns regarding search syntax modularity and result reproducibility. Studies published in French were screened and reviewed using the Google Translate service, which evidence suggests “is a viable, accurate tool for translating non–English-language trials for the purpose of conducting systematic reviews” [37]. A protocol for this review was registered on the Open Science Foundation’s open registries network [available at osf.io/ewjzk]. A sample of our full search strategy, as formatted for Ovid MEDLINE, Embase and Global Health, is shown in Table 1. Our search was conducted on the 17^th^ of September, 2022.

**Table 1:** Search strategy sample as formatted for Ovid MEDLINE, Embase and Global Health.

1	street children.mp. or exp Homeless Youth/ [mp = ti, bt, ab, ot, nm, hw, fx, kf, ox, px, rx, ui, sy, tn, dm, mf, dv, dq, cw]	2679
2	exp nigeria/ or Niger/	120792
3	1 and 2	52
4	(almajiri or almajirai).af.	29
5	("street child*" and "niger*").mp. [mp = ti, bt, ab, ot, nm, hw, fx, kf, ox, px, rx, ui, sy, tn, dm, mf, dv, dq, cw]	58
6	(homeless* and child* and niger*).mp. [mp = ti, bt, ab, ot, nm, hw, fx, kf, ox, px, rx, ui, sy, tn, dm, mf, dv, dq, cw]	33
7	(neglect* and child* and niger*).mp. [mp = ti, bt, ab, ot, nm, hw, fx, kf, ox, px, rx, ui, sy, tn, dm, mf, dv, dq, cw]	518
8	("out of school" and "child*" and "niger*").mp. [mp = ti, bt, ab, ot, nm, hw, fx, kf, ox, px, rx, ui, sy, tn, dm, mf, dv, dq, cw]	125
9	(enfant* and niger*).mp. [mp = ti, bt, ab, ot, nm, hw, fx, kf, ox, px, rx, ui, sy, tn, dm, mf, dv, dq, cw]	186
10	((quran* or koran* or qur’an* or kor’an*) and school* and niger*).mp. [mp = ti, bt, ab, ot, nm, hw, fx, kf, ox, px, rx, ui, sy, tn, dm, mf, dv, dq, cw]	23
11	(health* or disease* or illness* or sante* or maladie*).mp. [mp = ti, bt, ab, ot, nm, hw, fx, kf, ox, px, rx, ui, sy, tn, dm, mf, dv, dq, cw]	28323246
12	3 or 4 or 5 or 6 or 7 or 8 or 9 or 10	930
13	11 and 12	794
14	limit 13 to yr = "2000 -Current"	685

Titles and abstracts identified through our search strategy were screened by a team of seven reviewers such that each study was screened by two independent reviewers. Conflict resolution and full-text review were performed by two reviewers with previous experience conducting literature reviews, lived experience in Hausaland, and linguistic proficiency in Hausa. Data extraction was performed by the primary investigator. Specific pieces of information collected for each study included (i) author, (ii) study year and location(s), (iii) study design and aims, (iv) study sample characteristics, (v) key findings, and (vi) almajiri participation in research design, execution, interpretation, and dissemination.

## Results

**Fig 1:**
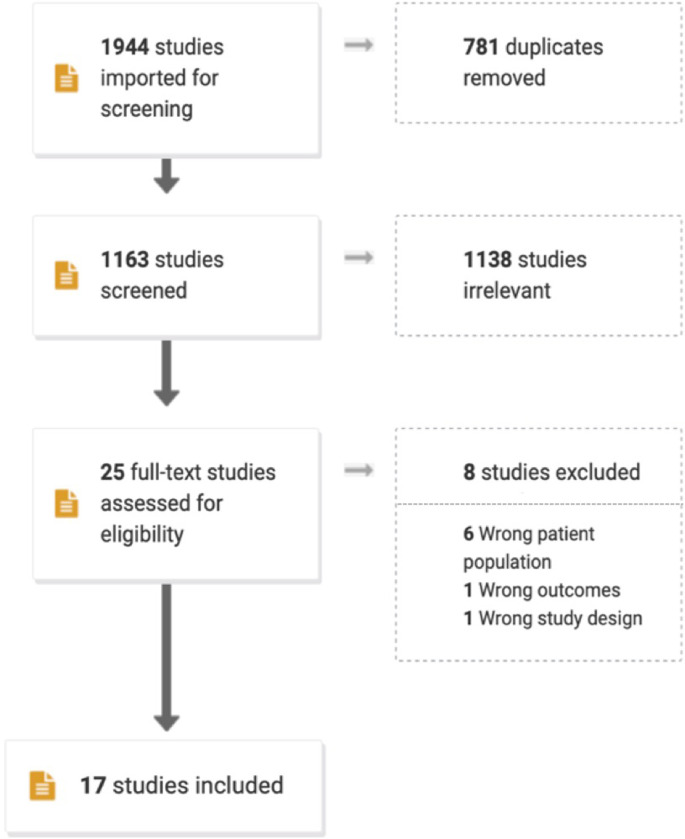
PRISMA flow-chart for included article. Of 1994 studies identified through our search strategy, 25 were included in the full-text review, and 17 were eligible for inclusion in this study.

Our search identified 1,944 studies across our six databases, of which 781 were duplicates. Of 1,163 studies included in title and abstract screening, 25 were included in full-text review. After full-text review, a final set of 17 studies were deemed relevant for data extraction. Of 8 studies included in full-text review but not in data extraction, 6 did not concern almajirai, one did not report health-related outcomes, and another did not present data. A Preferred Reporting Items for Systematic Reviews and Meta-Analyses (PRISMA) flow-chart depicting the outcomes of our screening and review processes is shown in Fig 1.

Our 17 studies included 14 cross-sectional studies, 2 descriptive articles, and one case-control study. Just one study concerned an intervention to improve almajiri health. No study engaged almajirai in participatory roles beyond acquiring their consent. All 17 studies were conducted in Nigeria, though one included almajirai from Niger living in Nigeria [31]. Studies reporting original data represented 6 of Northern Nigeria’s 19 states. These were Adamawa, Bauchi, Borno, Kaduna, Kano, and Sokoto.

While most studies focused on almajirai, or compared almajirai to control samples of secular school students, one study concerning almajirai employed as waste-pickers considered them alongside others engaged in this work and did not disaggregate data for specific groups [38]. The ages of almajirai represented in our review ranged from as young as 3 years to as old as 28, though some studies did not clearly state participants’ ages.

Table 2 shows the results of our data extraction process for our final set of 17 studies. These studies may be grouped into domains reflecting their content and focus. Ten articles concerned infectious disease, the most of any domain in this review, while other articles concerned such topics as workplace injury, nutrition, general health status, health determinants, oral health and mental health. We use these domains to present these studies and their main findings below.

**Table 2:** Final articles included in this review.

*Author (Year)*	*Title*	*Year*	*Location*	*Study Design*	*No. of Participants*	*Participant Ages*	*Main Findings*	*Notes on Participation*	*Other Notes*
***Dimas 2017***	The Drivers Of The Cholera Epidemic In Bauchi, Northeast Nigeria 2014	2014	Bauchi City, Bauchi State, Nigeria	Case control study assessing cholera risk factors around a 2014 Cholera outbreak in Nigeria’s North-East Bauchi State	124 cases and 124 controls. 26 cases and 6 controls were almajirai.	Not disclosed explicitly.	“Contact with a diarrhoea case, being an ‘Almajiri’ and unhygienic behaviors are major risks factors for the spread of the disease”	None disclosed. Recommend teaching hand washing to almajirai in Tsangayu.	
***Adeleke 2008***	Dermatophytosis Among Itinerant Quranic Scholars In Kano (Northwest) Nigeria	2006	Tarauni Local Government Area, Kano State, Nigeria	Cross-sectional study using physical exams and skin sample testing to determine the prevalence of dermatophytosis among almajirai	2,150 almajirai	Range: 5–25	Dermatophyte infections were seen in 9.5% of participants, though most infections were mild.	Consent obtained from mallamai. Recommend periodic skin examinations for dermatophytosis among almajirai.	
***Sarkingobir 2019***	Assessment Of Selected Health Determinants Among Almajiri Students In Gwadabawa Local Government, Sokoto State, Nigeria	Not explicitly disclosed	Gwadabawa Local Government Area, Sokoto, Nigeria	Cross sectional study using semi-structured questionnaires to evaluate school building quality, potable water access, safe toilet use, hygiene, nutrition and exposure to violence among almajirai	40 almajirai.	Range: 7–17	50% of almajirai schooled in mud- or corrugated-metal buildings. 66% of Tsangayu did not have close access to potable water, and 50% were situated around refuse dumps and open wastewater gutters.	None disclosed.	30 almajirai reported their citizenship as Nigerian, and 10 reported their citizenship as Nigerien.
***Damen 2011***	Prevalence Of Intestinal Parasites Among Pupils In Rural North Eastern, Nigeria.	2006	Konduga Local Government Area, Borno State, Nigeria	Cross-sectional study using stool sample testing to determine the prevalence of intestinal parasitosis among almajirai	257 almajirai	Range: 5–16	208 (80.9%) almajirai had intestinal parasites.	Ethical clearance and consent obtained from an Imam at a local mosque, and mallamai at each participating Tsangaya. Recommends ‘public engagement’ to prevent and control pediatric parasitic infections, but does not specify these for almajirai.	
***Sclama 2017***	Mainstreaming Nutrition In A School-Based Feeding Programme In Northeast Nigeria.	Not explicitly disclosed	Yola City, Adama State, Nigeria	Article about an American University of Nigeria (AUN)-designed intervention providing almajirai with daily meals, basic education and connection to vocational training	Total number of participants unclear, but article references plans to “reach over 1,000 students”, though this number may include girls who are not almajirai.	Not explicitly disclosed but describes almajirai as “typically between the ages of 6 and 25”	In 2016, an assessment of 200 boys graduated from the program “found that they had made a significant improvement based on the Early Grade Reading Assessment (EGRA) and Early Grade Mathematics Assessment (EGMA) exams.”	Mentions efforts to attain “of the children’s parents, guardians, mallams, other community members and state government officials” largely through the Adamawa Peace Initiative, a collaboration between the AUN and local religious leaders	No outcomes yet reported. Of note, program has also been extended to girls.
***Aminu 2017***	Carriage Rate Of Neisseria Meningitides Among Pupils Of Islamic Boarding Schools (Tsangaya Almajirai) In Kano, Nigeria.	2016	Kano State, Nigeria	Cross-sectional study using nasal sample testing to estimate the prevalence of Neisseria meningitides among almajirai	150 almajirai	Range: 5–10	23 (15.3%) almajirai had nasal samples that were positive for N. meningitides, around half of whom (52.2%) were positive for N. meningitides serotype B	Ethical clearance and consent for the study were obtained from the Kano State Board of Islamiyya and Quranic education, and from “head teachers” at each Tsangaya.	
***Hamma 2017***	Malaria Occurrence And Awareness Amongst Almajirai In Some Selected Traditional Qur’anic Centres Of Kano Municipal And Gwale Local Government Areas Of Kano State.	2017	Kano Municipal & Gwale Local Government Areas, Kano State, Nigeria	Cross-sectional study using blood sample microscopy to estimate the prevalence of malaria among almajirai, and speciate prevalent strains.	454 almajirai	Range: 9–28	162 (35.7%) almajirai had blood samples that were positive for malaria. P falciparum was the only malarial species represented in the sample.	Ethical approval was obtained from an Islamiyya school boards, and verbal consents were obtained from each participating almajirai. Authors recommend that “[c]ommunity mobilization and health education regarding importance of ITNs [insecticide-treated nets] should be considered”.	
***Shuaibu 2011***	Assessment Of Socioeconomic, Demographic And Health Problems Of Al-Majiri In Sokoto State, North-Western Nigeria	2011	Sokoto City, Sokoto State, Nigeria	Cross sectional study using semi-structured interviews, physical examinations and urine testing to “assess the demographic profile, socioeconomic backgrounds and health status” of almajirai in Sokoto state	377 almajirai	Not disclosed explicitly.	225 (59.7%) almajirai had features of urinary tract infection, out of which 116 (51.6%) yielded culture-positive E. coli, sensitive to siprosan, gentamycin and levoxin. 50 (13.3%) had dry skin and 40 (10.6%) had symptoms of upper respiratory infection. Skin lesions, diarrheal disease, eye discharge and bodily wounds were each seen in under 5% of the sample.	None disclosed.	
***Dzikwi 2012***	Knowledge, Attitude And Practice About Rabies Among Children Receiving Formal And Informal Education In Samaru, Zaria, Nigeria.	2010	Samaru Ward, Sabon Gari Local Government Area, Zaria City, Kaduna State, Nigeria	Cross sectional study using self- and interviewer-administered questionnaires to compare rabies knowledge and risk-related behaviors between children attending secular schools and almajirai	77 almajirai and 400 children from secular primary and secondary schools	Range: 5–20	203 (50.8%) children in secular schools and 25 (32.5%) almajirai had any knowledge about rabies. Of those who recognized rabies, 127 children in secular schools (65.7%) and 2 almajirai (8%) were aware that dog bites could transmit the disease.	Consent for the study was obtained from ‘responsible authorities’. Authors “recommend rabies education for parents and school teachers in both the formal and informal setting” for better recognition, prevention and treatment of rabies.	
***Akintunde 2020***	Public Health Implication Of Displacement Of Almajiri Children In Specific States Of Northern Nigeria Amidst COVID-19 Pandemic	2020	N/A	Text and opinion piece using descriptive analysis and “various sources such as government documents, websites, and blogs” to evaluate the efforts many Nigerian states undertook to repatriate almajirai to their communities of origin in the face of the COVID-19 pandemic.	N/A	N/A	States returned up to thousands of almajirai to their putative states of origin during the early phase of the COVID-19 pandemic, despite limited evidence of widespread COVID-19 infection among almajirai. Few if ay measures were taken to quarantine and provide care for almajirai who experienced this displacement.	N/A	
***Ali 2021***	Prevalence Of Injuries Among Waste Pickers. A Case Study In Nigeria	2019	Bauchi City, Bauchi State, Nigeria	Cross sectional study using questionnaires to estimate the prevalence of work-related physical injury, and commonly used means to treat these injuries, among waste-pickers in Bauchi city.	313 participants, of whom the number that were almajirai is not disclosed.	Not disaggregated for almajiri participants	246 respondents (78.5%) reported having experienced work-related injury. Reported injuries, in order of prevalence, were laceration (37.8%), musculoskeletal injury (23.7%), rashes (14%), animal bites (13.5%), piercing injuries (10.6%) and burns (8.3%). Almajirai and workers without experiences of secular education were more likely to report having experienced injury. 54% of waste pickers do not pursue medical treatment for wound, and instead pursue alternative means of management. Such means included the use of procaine powder, ash, sand, salt, grass fluid, hydraulic, kerosene, battery acid, and herbal medicines.	None disclosed.	Authors note that “most of those waste pickers are Internally Displaced Persons (IDPs) and Almajiri children”. Results are not, however, disaggregated by worker type.
***Balogun 2016***	Asymptomatic Falciparum Malaria And Genetic Polymorphisms Of Pfcrt K76T And Pfmdr1 N86Y Among Almajirai In Northeast Nigeria	2010	Maiduguri City, Borno State, Nigeria	Cross sectional study using blood sample testing to estimate the prevalence of asymptomatic Plasmodium falciparum malaria, as well as malarial mutations conferring resistance to chloroquine, among almajirai.	440 almajirai	3–12 (Range)	The prevalence of asymptomatic falciparum parasitemia and gametocytemia were 12.7% (56/440) and 8.6% (38/440), respectively. Among almajirai with parasitemia, 3 (5.4%) had one of two malarial mutations conferring chloroquine resistance (Pfcrt K76T). Another resistance-conferring mutation (Pfmdr1 N86Y) was not seen in this sample.	Ethical approval and research permission was obtained from the Borno State Ministry of Religious Affairs, and consent was obtained from participating almajirai.	
***Gambo 2021***	A Comparative Study On The Prevalence And Intensity Of Urinary Schistosomiasis Among Primary (Formal) And Almajiri (Informal) School Pupils In Kura Local Government Area Of Kano State, Nigeria	Not explicitly disclosed	Kura Local Government Area, Kano State, Nigeria	Cross sectional study using urine sample testing to compare the prevalence of urinary schistosomiasis between almajirai and secular primary school students.	200 almajirai and 200 secular primary school students	Mean: 10.7 (Almajirai) Mean: 10.3 (Secular)	Urinary schistosomiasis was seen in 86 secular school students (43%) and 111 almajirai (55.5%), a statistically significant difference (p<0.05).	Written informed consent signed or thumb printed was obtained from parents, guardians or mallamai. Older pupils aged 7 years and above gave their assent. Authors “recommend that control programmes should target more on Almajiri school pupils in addition to the primary school pupils."	
***Yandoma 2019***	Risk Factors For Intestinal Parasitosis Among Almajiri Pupils In Zaria, North Western Nigeria	Not explicitly disclosed	Zaria City, Kaduna State, Nigeria	Cross sectional study using questionnaires and stool samples to evaluate the prevalence of intestinal parasitosis among almajirai, as well as risk factors associated with parasitic infection.	262 almajirai	Range: 4–12	218 almajirai (83.2%) had intestinal parasites. Intestinal parasitosis was significantly associated with older age, poor hand washing after defecation, fingernail-biting and thumb-sucking, sharing food from a plate and belonging to a polygamous family (p<0.05).	Permission for the study was obtained from the local authority overseeing the schools. Assent for participation was obtained from individual pupils and consent obtained from their mallamai. Authors recommend that "proper sewage disposal and personal and environmental hygienic practices should be inculcated in the Almajiri system."	
***Abubakar-Abdullateef 2017***	A Comparative Study Of The Prevalence And Correlates Of Psychiatric Disorders In Almajiris And Public Primary School Pupils In Zaria, Northwest Nigeria	Not explicitly disclosed	Zaria City, Kaduna State, Nigeria	Cross sectional study using the Schedule for Affective Disorders and Schizophrenia for School aged Children- Present and Lifetime Version (K-SADS-PL), a validated semi-structured diagnostic interview tool, to compare the prevalence of psychiatric disorders between almajirai and secular primary school children.	213 almajirai and and 200 students at secular public primary schools	Range: 5–19 Mean: 13.1 (Almajirai) Mean: 10.9 (Secular)	The prevalence of depression, general anxiety disorder, enuresis, substance use and PTSD were significantly higher among almajirai than among students at secular schools. Almajirai were significantly less likely than secular school students to have separation anxiety. Significant associations were found between psychiatric diagnoses and maternal education, personal experience of malnutrition, serious injury, fighting, bullying within the last month and visiting home less than 3 times per year.	Consent for study was obtained from mallamai, and assent was obtained from all participating almajirai.	The mean ages in the almajiri and secular school groups were significantly different in this study (p<0.05).
***Idowu 2016***	Oral Health Knowledge And Practice Of 12 To 14-Year-Old Almajaris In Nigeria: A Problem Of Definition And A Call To Action	Not explicitly disclosed	Nasarawa Local Government Area, Kano State, Nigeria	Cross sectional study using a questionnaire to assess oral health knowledge, behaviors and medical history among almajirai, and a simplified Oral Hygiene Index (OHI-S) to evaluate their oral hygiene.	186 almajirai	Mean: 12.7	104 almajirai (56%) reported practicing oral hygiene to prevent mouth odor and 12 (6.4%) practiced oral hygiene to prevent dental caries and periodontal disease. 6 per cent practiced oral hygienemeasures for the prevention of dental caries and periodontal diseaseas well. 156 (84%) reported daily teeth cleaning. 67% of almajirai reported using water and a finger to clean their teeth, with 4% using toothpaste and a toothbrush, and 29% using either a toothbrush or chewing stick without toothpaste. The OHI-S revealed ‘good’ oral hygiene for 2 almajirai (1%), with the remaining 184 (99%) equally split between ‘poor’ and ‘fair’ oral hygeine. Just 3% of respondents were familiar with dental floss and 2% aware of the need for regular dental visits to maintain oral health.		
***Idowu 2020***	Nigeria’s Street Children, Epitome Of Oral Health Disparity And Inequality	2020	Nasarawa Local Government Area, Kano State, Nigeria	Cross sectional study using a questionnaire and simplified Oral Hygiene Index (OHI-S) to compare oral health knowledge, behaviors, and oral hygiene between almajirai and students at private and secular secondary schools.	200 almajirai and 200 students at private secular secondary schools	Mean: 12.7 (Almajirai) Mean: 13.05 (Secular)	6% of almajirai and 70% of secular students identified that oral hygiene prevents both mouth odor and oral disease. 5% of almajirai and 90% of secular students used a toothbrush and toothpaste for teeth cleaning. 65% of almajirai performed teeth cleaning with their fingers and water alone. 4 almajirai (2%) and 128 secular students (64%) had ‘good’ oral health on the OHI-S.	Consent for study was obtained from mallamai. Authors recommend that “both the mallams and the secondary school teachers should be specially educated on oral hygiene practices” and that “oral health care delivery should be made more accessible to the Nigerian children through the establishment of mobile dental clinics that will pay periodic visits to Quranic and formal schools”	

### Infectious disease

#### Malaria

Hamma et al., in a 2017 study conducted in the Kano Municipal and Gwale Local Government Areas of Nigeria’s Kano State, used blood microscopy in a sample of 454 almajirai, aged 9 to 28 years old, to assess the prevalence of malaria and speciate prevalent strains. The prevalence of malaria in this sample was 35.7%, and P. falciparum was the only malarial species found in the sample. The authors noted that estimates of malaria prevalence among other groups in Northern Nigeria have been similar or lower than the prevalence seen for almajirai (30.6%, 36.5% and 19.3%), and recommended that “community mobilization and health education regarding importance of ITNs [insecticide-treated nets] should be considered” [39].

Balogun et al., in their 2010 study based in Maiduguri, Borno State, hypothesized that almajirai’s poor shelter disproportionately exposes them to mosquito bites, and that this exposure may induce pre-immunity to, and asymptomatic infection with falciparum malaria. They further considered trends in chloroquine resistance in Nigeria, and used blood microscopy and genetic testing, respectively, to determine the prevalence of asymptomatic malarial infection and two malarial mutations conferring resistance to chloroquine among a sample of 440 almajirai. Almajirai in the sample ranged from 3 to 12 year of age. The prevalence of asymptomatic falciparum parasitemia and gametocytemia were 12.7% (56/440) and 8.6% (38/440), respectively. Among almajirai with parasitemia, 3 (5.4%) had one of two malarial mutations conferring chloroquine resistance (Pfcrt K76T). Another resistance-conferring mutation (Pfmdr1 N86Y) was not seen in this sample. These authors argued that almajirai “should be incorporated into various malaria control programs to ensure the success of the fight against malaria in Nigeria at large” [40].

#### Intestinal parasitosis

Damen et al., in a 2006 study in Borno State’s Konduga Local Government Area, used stool sample testing to determine the prevalence of intestinal parasitosis among 257 almajirai, and found that 208 (80.9%) had intestinal parasites, which the authors noted as higher than the prevalence seen in studies in Nigeria’s Southwest, Southeast and South-south regions (28%, 55.2%, and 67.2%, respectvely). Similarly, Yandoma et al. (2019) used stool microscopy and a health behavior survey to assess the prevalence of, as well as risk factors for, intestinal parasitosis in a sample of 262 almajirai from Zaria city in Kaduna state. This study found that 218 almajirai (83.2%) had intestinal parasites, and that intestinal parasitosis was significantly associated with older age, poor hand washing after defecation, fingernail-biting and thumb-sucking, sharing food from a plate and belonging to a polygamous family (p<0.05). The authors recommended ‘public engagement’ to prevent and control pediatric parasitic infections, but did not specify this for almajirai [41].

#### Other infectious disease

Dimas et al. (2017) evaluated risk factors associated with cholera infection following a 2014 outbreak of cholera in Maiduguri, Borno State in a case-control study. This study’s sample included 124 cases and 124 controls, of whom 26 cases and 6 controls were almajirai. Their evaluation found an odds ratio (OR) of 5.2 (Confidence Interval [CI] 1.94–14.78) for cholera infection among almajirai, and the authors recommended that almajirai be taught effective hand washing techniques at their Tsangayu to help prevent and control cholera [14].

Adeleke et al. (2008), in a 2006 study of 2,150 almajirai in Kano State’s Taurani Local Government Area, argued that the crowded conditions in which many almajirai live and learn predispose almajirai to dermatophytosis, and used physical exams and mycological studies to evaluate the presence of dermatophytosis among almajirai. This study found dermatophytosis in 9.5% of participants, reporting that “most infections were mild”, and noted that this prevalence is similar to that seen for secular primary school children in North-central Nigeria, but lower than that observed in a hospital study in Eastern Nigeria (7.8% and 20.8%, respectively). These authors argued for periodic skin examinations to evaluate dermatophytosis among almajirai [42].

Aminu et al. (2017) considered the prevalence of Neisseria meningitidis in a sample of 150 almajirai, aged 5 to 10, from Kano State, and used nasal sample tests to determine this prevalence and speciate prevalent strains. They found that 23 (15.3%) almajirai had nasal samples that were positive for N. meningitides, around half of whom (52.2%) were positive for N. meningitides serotype B [43]. The authors noted that the prevalence of N. meningitidis seen here was higher than that seen in studies of other populations in Northern Nigeria (7.4%).

Gambo et al. (2021) used urine microscopy to compare the prevalence of urinary schistosomiasis between 200 almajirai and 200 secular primary school students in Kura Local Government Area, Kano State. The mean ages in these groups were 10.7 and 10.3 years, respectively. Urinary schistosomiasis was seen in 86 secular school students (43%) and 111 almajirai (55.5%), a statistically significant difference (p<0.05). Here, authors “recommend that [schistosomiasis] control programmes should target … Almajiri school pupils in addition to the primary school pupils" [15].

In another comparative study, Dzikwi et al. (2012) used self- and interviewer-administrated surveys to compare rabies knowledge and risk-related behaviors between almajirai and children attending secular schools. Their sample included 77 almajirai and 400 children in secular primary and secondary schools from the Samaru Local Government Area in Zaria City, Kaduna State, with ages between 5 and 20 years. They found that 203 (50.8%) children in secular schools and 25 (32.5%) almajirai had any knowledge about rabies. Of those who recognized rabies, 127 children in secular schools (65.7%) and 2 almajirai (8%) were aware that dog bites could transmit the disease. These authors “recommend rabies education for parents and school teachers in both the formal and informal setting” for better recognition, prevention and treatment of rabies [44].

Akintunde et al. (2020) considered anxieties around almajiranci in the context of the COVID-19 pandemic in Nigeria, where almajirai’s mobility and exposure to communicable diseases caused them to be seen as particularly vulnerable to viral infection and transmission. These anxieties caused some state governments to consider repatriating almajirai to their places of origin, to limit the risk they perceived almajirai as bringing to their host states. This study performed descriptive analysis on “various sources such as government documents, websites, and blogs” to evaluate if and how this repatriation occurred, and consider its impact on almajirai and Nigerian public health more broadly. They found that various states returned up to thousands of almajirai to their putative states of origin during the early phase of the COVID-19 pandemic, despite limited evidence of widespread COVID-19 infection among almajirai. Few if any measures were taken to quarantine and provide care for almajirai who experienced this displacement. The authors used this evidence to argue that repatriation “may not be the best course of action because it will increase the possibility of infecting people with the virus”, and that a more effective means of management may be for governments to establish camps for almajirai in their host states with “shelter for the children, provision of food and ultimately medical care” [17].

### Other topics

#### Oral health

Idowu et al. (2016) used a questionnaire to survey 186 almajirai in Kano State’s Nasawara Local Government Area about their oral health knowledge, behaviors and dental history, and a simplified Oral Hygiene Index (OHI-S) to evaluate their oral hygiene. They found that 104 almajirai (56%) reported practicing oral hygiene to prevent mouth odor and 12 (6.4%) practiced oral hygiene to prevent dental caries and periodontal disease. Additionally, while 156 (84%) almajirai reported daily teeth cleaning, just 4% of this figure reported using a toothbrush and toothpaste, with the remainder using their fingers, chewing sticks or toothbrushes with water. The OHI-S revealed ‘good’ oral hygiene for 2 almajirai (1%), with the remaining 184 (99%) equally split between ‘poor’ and ‘fair’ oral hygiene. Just 3% of respondents were familiar with dental floss and 2% aware of the need for regular dental visits to maintain oral health [45].

These same authors, in a 2020 study, compared oral health behaviors and oral hygiene between almajirai and students attending private secondary schools. Their sample consisted of 200 almajirai and 200 secondary school students, with mean ages of 12.7 and 13.05 years, respectively. This analysis found that 6% of almajirai and 70% of PSS students identified that oral hygiene prevents both mouth odor and oral disease, 5% of almajirai and 90% of secondary school students used a toothbrush and toothpaste for teeth cleaning, and that 4 almajirai (2%) and 128 secondary school students (64%) had ‘good’ oral health on the OHI-S. Here, authors recommended that “both the mallams and the secondary school teachers should be specially educated on oral hygiene practices” and that “oral health care delivery should be made more accessible to the Nigerian children through the establishment of mobile dental clinics that will pay periodic visits to Quranic and formal schools” [46].

#### Nutrition

Sclama (2017) considered an intervention to improve nutrition for almajirai in Yola, the capital of Nigeria’s Adamawa State. This intervention, designed and implemented by the American University of Nigeria (AUN), based in Yola, provided almajirai with daily meals, basic education and connection to vocational training. The program’s total number of participants was not stated explicitly, but this article referenced plans to “reach over 1,000 students”. Though growth- and nutrition-related outcomes of this intervention have not yet been reported, an assessment of 200 boys graduated from the program “found that they had made a significant improvement based on the Early Grade Reading Assessment (EGRA) and Early Grade Mathematics Assessment (EGMA) exams”. The article further discussed efforts to gain support for the program from “children’s parents, guardians, mallams, other community members and state government officials” through the Adamawa Peace Initiative, a collaboration between the AUN and local religious leaders. Of note, while this intervention was initially designed for almajirai, by the time of the article’s writing, it had been extended to include girls who are not almajirai but demonstrate similar needs [47].

#### Health determinants

Sarkingobir et al. (2019) used a semi-structured survey to evaluate a broad range of environmental health determinants in a sample of 40 almajirai, ages 7 to 17, in Sokoto State’s Gwadabawa Local Government Area. Their study found that 50% of almajirai schooled in mud- or corrugated-metal buildings, 66% of Tsangayu did not have close access to potable water, and 50% were situated around refuse dumps and open wastewater gutters. Of note, in this sample, 30 almajirai reported their citizenship as Nigerian, and 10 reported their citizenship as Nigerien [31].

#### General health status

Shuaibu et al. (2011) used semi-structured interviews, physical examinations and urine testing to “assess the demographic profile, socioeconomic backgrounds and health status” of 377 almajirai in Sokoto, the capital city of Sokoto State. They found that 225 (59.7%) almajirai had features of urinary tract infection, out of which 116 (51.6%) yielded culture-positive E. coli on urine testing, that was sensitive to siprosan, gentamycin and levoxin. 50 (13.3%) had dry skin and 40 (10.6%) had symptoms of upper respiratory infection. Skin lesions, diarrheal disease, eye discharge and bodily wounds were each seen in under 5% of the sample [48].

#### Work-related injury

Ali et al. (2021), in a 2019 study set in Bauchi city, Bauchi State, considered workplace injury among people employed in waste-picking, which “involves the collection, purchase, and recovery of materials for economic benefit” from waste and refuse dumps. Here, authors identified that, of their 313 participants, most were “Internally Displaced Persons (IDPs) and Almajiri children”, but did not disaggregate their findings by participant type. They found that 246 respondents (78.5%) reported having experienced work-related injury. Reported injuries, in order of prevalence, were laceration (37.8%), musculoskeletal injury (23.7%), rashes (14%), animal bites (13.5%), piercing injuries (10.6%) and burns (8.3%). Almajirai and workers without experiences of secular education were more likely to report having experienced injury. 54% of respondents reported not pursuing medical treatment for wounds, and instead pursued alternative means of management. Such means included the use of procaine powder, ash, sand, salt, grass fluid, hydraulic, kerosene, battery acid, and herbal medicines. These authors recommended that waste-pickers receive “medical check-ups and immunization against tetanus and other … infections”, that the middlemen to whom they sell recovered materials provide waste-pickers with PPE, that local governments take measures to protect waste-pickers by separating hazardous waste from other types of waste, and that waste-pickers “form a union in order to protect their labor rights” [38].

#### Mental health

In a 2017 study set in Zaria city, Kaduna State, Abubakar-Abdullateef et al. (2017) used the Schedule for Affective Disorders and Schizophrenia for School aged Children-Present and Lifetime Version (K-SADS-PL), a validated semi-structured diagnostic interview tool, to compare the prevalence of psychiatric disorders between almajirai and secular primary school children. Their sample consisted of 213 almajirai and 200 public school students, aged 5 to 19. These authors used multivariate logistic regression to find significantly higher odds of depression (OR 2.93, CI 1.27–6.76), enuresis (OR 3.42, CI 1.59–7.36), substance use (OR 10.05, CI 1.20–84.06), post-traumatic stress disorder (OR 6.20, CI 1.90–20.19), and any psychiatric diagnosis (OR 3.11, CI 1.79–5.41) among almajirai than among students at secular schools (p<0.05). The odds of general anxiety disorder were significantly higher for almajirai in a univariate model, but were not significantly different from the odds observed for secular school students in the full multivariate model (OR 2.16 vs. 1.92, CI 1.15–4.05 vs. 0.79–4.70). Almajirai also had significantly lower odds than secular school students to have separation anxiety in the multivariate model (OR 0.14, CI 0.03–0.64; p<0.05). Among almajirai, significant associations were found between psychiatric diagnoses and poor maternal education, personal experience of malnutrition, serious injury, fighting, bullying within the last month and visiting home less than 3 times per year. Of note, the mean age among almajirai was significantly higher than the mean age for secular school students (13.1 vs 10.9; p<0.05). Authors here recommended “efforts towards improving [almajirai’s] socio-economic status and providing them with formal education”, government action to “promote physical and mental health, including general health education, screening, early detection and management of pupils at risk of developing psychiatric disorders”, and the abolition of migration as a feature of almajiranci, which the authors argue diminish “the protective family unit and parent–child interaction”, making almajirai more vulnerable [16].

## Discussion

This review highlights several notable features of the literature around almajiri health. One particularly striking finding is the limited geographic area represented in this literature. No study was conducted in Niger, and Northern Nigerian representation was limited to just 6 of that region’s 19 states. Additionally, just two Northern Nigerian states, Kano and Kaduna, made up over half of all studies identified in this review. All this comes despite almajiranci being a region-wide phenomenon, in practice throughout Hausaland in Nigeria and Niger, and one where almajirai’s intraregional migration remains a key feature. We see some of the true range of this system in that one study’s sample of almajirai included ten who reported their citizenship as Nigerien. While this type of inclusion is encouraging, much more study is needed that includes almajirai throughout their true geographic scope, to more fully represent their experiences in the different spaces in which they live, work and learn.

Another finding of note here is the wide range of represented ages. Almajirai included in these studies ranged from as young as 3 to as old as 28 years, representing individuals in both very early childhood and established adulthood. This large difference in age may further reflect profound differences in experience, perspective and priorities, each of which requires intentional analysis to not go unseen. Some studies with samples with particularly large age differences did evaluate outcomes by age, finding, for example, higher prevalence of dermatophytosis among almajirai in their early teenage years than in other age groups, and greater burdens of intestinal parasitosis among younger almajirai than among their older peers. These findings suggest that future studies around almajiri health may benefit from making more explicit the reasons that inform the ages of almajirai they choose to recruit, as well as disaggregating outcome measures by age.

We find that infectious disease studies and studies using cross-sectional designs made up particularly large portions of the literature around almajiri health. The number of cross-sectional designs seen here may reflect the relative inexpensive of such research designs, as well as the value these add to the nascent literature around almajiri health in providing evidence for specific health problems in this population [49]. The preponderance of infectious disease among these studies may reflect broader trends in the global health literature, where communicable disease has historically been a major area of research and health investment, as well as the heightened exposure to communicable diseases and their vectors that many believe exist among almajirai. We see this belief reflected in the studies identified here, with authors arguing that almajirai’s “overcrowded living conditions … easily allow the dissemination of microorganisms” and that the “poor hygienic practice and sanitary environment” in which almajirai live predispose them to contracting and transmitting infectious disease [41, 43].

Despite the pervasiveness of such beliefs, just three studies compare infectious disease among almajirai to control samples, finding higher rates of cholera and urinary schistosomiasis, as well as lower levels of rabies awareness among almajirai. Beyond infectious disease, two studies use control samples to find higher rates of psychiatric disorders and poorer oral hygiene in almajirai than in controls. While some other studies that do not include control samples in their designs compare their findings for almajirai to those seen for other populations in similar contexts, discussing, for example, differences in the prevalence of malaria, N. meningitidis and dermatophytosis, the lack of means to assess the direct comparability of these groups to almajirai limits the utility of these comparisons.

Additionally, though studies that make use of control groups provide valuable insights into health disparities for almajirai, we find considerable variety in the types of groups these studies select as controls. Of four cross-sectional studies with control groups, we find two using either public or private school students as controls, and two that did not specify the types of schools from which their control samples were obtained. While mean ages for almajirai and control groups were significantly different just one of these studies, the previously discussed differences between private and public schools in Northern Nigeria, where private schools’ fees have excluded poorer children, may be cause for some concern about the comparability of these studies’ findings. Comparisons to well-resourced students in private schools may exaggerate the true size of disparities between almajirai and general populations in the places they live, while comparisons to students in public schools, who may also experience socioeconomic deprivation, may impede study of best-case outcomes for health among almajirai. Future research around almajiri health may find benefit in making reasoning around control group selection more explicit, to allow better assessment of how research findings reflect reality.

Another finding of interest is that just two outcomes––oral hygiene and intestinal parasitosis––were evaluated in more than one study. Findings for these outcomes were largely consistent between studies, with the prevalence of intestinal parasitosis estimated at around 80% in two studies, and similar trends in almajirai’s oral hygiene behaviors, knowledge and outcomes seen in another two studies. This agreement is encouraging, and suggests that the findings of research around almajiri health may accurately reflect real conditions, though more study is needed that demonstrates this kind of congruence in other areas of concern.

We further note a dearth of research considering interventions to improve health among almajirai, and a complete absence of almajiri participation in helping to guide goals, strategies and interpretation in the study of their health, with almajiri participation in all studies limited to their provision of consent. Just one study identified in this review concerned a health intervention, providing almajirai in Yola city daily hot meals and connection to educational and vocational opportunities. While this study provides evidence that this intervention has helped improve educational attainment among almajirai, health and nutritional outcomes associated with this intervention have not yet been reported. This study, too, though referencing strategies to gain support for the program from almajirai and members of their communities, makes no mention of any input from these partners on the intervention’s design, and does not state explicitly what strategies have been used to develop these partnerships.

While the lack of study around interventions to improve almajiri health may reflect the fledgling state of this literature, the lack of participatory approaches to this research is somewhat more concerning. As previously mentioned, participatory approaches hold great promise for delivering sustainable solutions to community health problems, and, outside of health research, participatory work with almajirai has demonstrated this group’s enthusiasm and capacity for meaningful contribution to the discourse around their experiences [19, 20]. Additionally, 11 of the 17 studies included here, while not using participatory approaches themselves, stress the need for future work directly engaging almajirai to improve their health. Participatory approaches may be of particular importance in Hausaland, where distrust of modern medical practice remains widespread, and where partnership between health researchers and local communities may help navigate this distrust. That many almajirai are not just young children but adolescents and even young adults strengthens prospects for participation, as these older students may be more able to place their experiences in context by virtue of their greater maturity. This should, however, certainly not exclude younger almajirai from participatory roles, as input from almajirai at all ages enriches research, allowing insights into their perspectives and aspirations across these ages, as well the factors that influence how these may change over time.

We suggest that future research around almajiri health may find value in the adoption of participatory approaches. Participatory work may, for example, be used to develop interventions for known health disparities that almajirai face, such as in infectious disease, oral health and mental health, evaluate potential disparity for conditions highly prevalent among almajirai, such as intestinal parasitosis, or pursue new areas of health research of particular concern to almajirai and their communities, altogether strengthening this field of research. These approaches, beyond holding the promise outlined above, may help inform and strengthen the literature as research moves from data-gathering work to interventions meant to improve almajiri health, ensuring that these interventions benefit from the insight of the communities they intend to support. Participatory approaches would also help build partnerships between almajirai and the research enterprise, creating opportunities for longitudinal relationships between researchers and almajirai, and more intuitive transitions between data-gathering and intervention-deployment phases of research. Work is needed that outlines effective strategies to develop these types of partnerships with almajirai, their mallamai, and their communities more broadly, and that highlights potential areas of conflict that may arise in these relationships. All of these may help develop a common set of ethical standards for research around almajiri health that strengthens this research while protecting and advancing almajirai’s interests in society.

### Limitations

The major limitation of this work is a lack of representation of studies in the grey literature. A considerable amount of research in Low- and Medium-Income Countries, such as most of the countries on the African continent, faces barriers to publication in the peer reviewed literature, with limited access in many of these contexts to the resources, financial and otherwise, required to disseminate research findings in this literature, and so may be limited to publication as theses and other formats in the grey literature [50]. Though we initially intended to include Google Scholar among our literature databases to include sources from the grey literature, a lack of search syntax options to narrow down search results, and general concerns regarding search reproducibility prevented our use of this database. Future study around the almajiri health literature may benefit from recruiting research personnel to systematically work through and include sources from this database, to better represent potential studies in the grey literature.

## Conclusion

Our scoping review, the first review of any kind around almajiri health, identifies several key findings in this area of research. We find that this research has concerned a wide range of domains, including infectious disease, workplace injury, nutrition, general health status, health determinants, oral health and mental health. Despite this breadth, the number of studies concerned with specific phenomena within each domain remains limited. We further note limitations in the geographic scope of the current literature around almajiri health, in the study of interventions meant to improve almajiri health, and in consideration of demographic features, such as age, that may influence almajirai’s experiences and health. We stress the need for further study in all these areas, and for participatory approaches to this study, which, by involving almajirai in the research process, can help develop trust between almajirai and the research enterprise, build interventions tailored to their priorities and preferences, and may be more likely to sustainably and successfully improve almajiri health and wellbeing.

## Supporting information

S1 Checklist Preferred Reporting Items for Systematic reviews and Meta-Analyses extension for Scoping Reviews (PRISMA-ScR) checklist. (PDF)
